# Effects of Dietary Astaxanthin on Growth, Coloration, Immunity, and Antioxidant Capacity in *Macrobrachium Rosenbergii*

**DOI:** 10.1155/anu/8865839

**Published:** 2025-06-13

**Authors:** Huarong Li, Lingyun Yu, Jie Wei, Bai Liufu, Qiyao Su, Kunhao Hong, Qiaoyan Zhou, Huaping Zhu, Yakun Wang

**Affiliations:** ^1^Key Laboratory of Tropical and Subtropical Fishery Resource Application and Cultivation of Ministry of Agriculture and Rural Affairs, Pearl River Fisheries Research Institute, Chinese Academy of Fishery Sciences, Guangzhou 510380, China; ^2^College of Fisheries and Life Science, Shanghai Ocean University, Shanghai 201306, China; ^3^School of Fishery, Zhejiang Ocean University, Zhoushan 316000, China; ^4^College of Fisheries, Jimei University, Xiamen 361021, China

**Keywords:** astaxanthin, body color, gene expression, growth performance, immunity, *Macrobrachium rosenbergii*

## Abstract

This study investigated the effects of astaxanthin (Ax) on the growth, body color, and immunity of *Macrobrachium rosenbergii*. Four different concentrations of Ax (0, 50, 100, and 200 mg kg^−1^) were supplemented in the basal diet to assess their impact on the growth, coloration, immunity, and antioxidant capacity in *M. rosenbergii*. After 10 and 20 weeks of feeding, compared to the control group, the addition of 100 mg kg⁻^1^ of Ax significantly increased the weight gain rate (WGR) and specific growth rate (SGR; *p* < 0.05) and shortened the molting cycle (*p* < 0.05). Addition of Ax increased the redness (*a*^⁣^*∗*^^ value) and yellowness (*b*^⁣^*∗*^^ value) of live and cooked *M. rosenbergii* (*p* < 0.05), and the muscle Ax content increased as the amount of added Ax increased. The enzyme activity results showed that after 20 weeks of feeding, the inclusion of 200 mg kg^−1^ of Ax significantly increased the activities of superoxide dismutase (SOD) and alkaline phosphatase (AKP) in *M. rosenbergii* (*p* < 0.05) and significantly decreased the activity of malondialdehyde (MDA; *p* < 0.05). Compared with control group, the addition of 100 mg kg^−1^ of Ax significantly increased the expression levels of genes related to immunity and antioxidant activity (*p* < 0.05). Additionally, there were significant differences in the expression levels of molting-related genes (ecdysteroid receptor (*EcR*) and retinoid X receptor (*RXR*)), across the different Ax supplementation levels and molting stages. Considering all factors, the addition of 100 mg kg^−1^ of Ax to the feed not only improved the growth performance and body color of *M. rosenbergii* but also enhanced its immune and antioxidant capabilities. This study provides a reference for the application of Ax in crustacean feed.

## 1. Introduction

Astaxanthin (Ax), also known as shrimp yellow or xanthin, is a lipid-soluble ketonic carotenoid widely found in aquatic animals and microorganisms [1–3]. There are two types of Ax: natural and synthetic. The former is extracted from organisms such as algae, yeast, and bacteria [4–7], whereas the latter is obtained through chemical synthesis [8]. Many aquatic animals, although they may contain Ax, these animals cannot synthesize it endogenously, and instead accumulate it in their bodies after ingesting it from food [9, 10]. Ax is a high-value ketonic carotenoid that is poorly soluble in water and acts as a potent natural antioxidant and achieves highly effective antioxidant function primarily by capturing free radicals on the surface and within phospholipid membranes [11–14]. It is commonly used to enhance the body's immunity, disease resistance, and stress tolerance, allowing it to better withstand various environmental stressors [15–18]. When 102.5 mg kg^−1^ of dietary Ax from *Phaffia rhodozyma* was supplemented to the feed of *Penaeus monodon* culturing for 56 days, the weight gain rate (WGR) increased by 27.62% and significant changes in immune and antioxidant enzyme activities, demonstrating that Ax can enhance the growth performance and improve the stress resistance [19]. Additionally, supplementing the feed of red swamp crayfish (*Procambarus clarkii*) with 0.6% Ax improved the stability of intestinal microbial community and alleviated immune damage in their bodies [20]. Similarly, the addition of Ax to the feed of white shrimp (*Litopenaeus vannamei*) not only improved its growth performance but also enhanced its immune and antioxidant capabilities [21].

In addition to these functions, Ax exhibits excellent coloring properties [22–24]. For example, in *Oncorhynchus mykiss* farming, as the Ax content increases, the redness (*a*^⁣^*∗*^^ value) of rainbow trout fillets gradually intensifies [25]. In ornamental fish cultivation, Ax is frequently incorporated into feed to alter body color. For example, adding natural Ax to the feed of discus fish (*Symphysodon haraldi*) [26] and koi carp (*Cyprinus carpio L*.) [27] can significantly elevate their redness (*a*^⁣^*∗*^^ value) and yellowness (*b*^⁣^*∗*^^ value), resulting in visually appealing coloration. Furthermore, Ax not only alters its content in muscles, it also shortens the molting cycle. After feeding *Exopalaemon carinicauda* with Ax-containing feed for 56 days, the shrimp's body surface noticeably turned redder, and both the total and free Ax contents in the abdominal muscle increased [28]; the addition of 80 mg kg^−1^ of Ax to the feed of juvenile *L. vannamei* shrimp significantly shortened their molting interval [29]. A recent study showed that dietary supplement of excessive Ax (320 mg kg^−1^) inhibited some molting related-gene expression, which has negative effect on the molting and metamorphosis of *M. rosenbergii* larvae [30].

The giant freshwater prawn (*Macrobrachium rosenbergii*) is an aquaculture species that is easy to feed, grows quickly, produces favorable tasting meat, and has a high nutritional value [31, 32]. According to the China Fishery Statistical Yearbook, the annual production of *M. rosenbergii* in China will exceed 190,000 tons by 2023 [33]. The farming of *M. rosenbergii* has not only generated income and increased efficiency for aquaculture farmers but has also promoted the development of China's agricultural economy. Currently, although several studies have reported that supplement of Ax to the feed could promote an increase in carotenoid content of *M. rosenbergii* [34]. Feeding *M. rosenbergii* with appropriate Ax-containing feed enhanced their growth and immune performance [35] and promote their metamorphosis [30], information about the effects of Ax on *M. rosenbergii* are still limited. Therefore, this study examined the impacts of feeding *M. rosenbergii* various doses of natural Ax on its growth, immunity, antioxidant enzyme activity, and molting cycle. We speculate the addition of natural Ax will promote growth, improve immunity, antioxidant capacity, and shorten molting cycle. The results will provide insights and guidance for the utilization of Ax in healthy aquaculture of *M. rosenbergii*.

## 2. Materials and Methods

### 2.1. Experimental Diets

The experimental base feed was obtained from Zhuhai Huolibao Feed Co., Ltd. (Zhuhai, Guangdong, China). After crushing and passing through a 60-mesh sieve, the feed ingredients were gradually mixed according to the feed formula (Table 1). They were then pelletized into hard and sinking feed particles with a diameter of 1.2 mm using a single-screw extruder (pelletizing temperature of 85 ± 5°C). The pellets were dried in a blast dryer at 40°C until the moisture content was below 10%, sealed, and stored at −20°C for future use. The natural Ax levels in the four feed groups were 0 mg kg^−1^ (Group C), 50 mg kg^−1^ (Group Ax50), 100 mg kg^−1^ (Group Ax100), and 200 mg kg^−1^ (Group Ax200).

### 2.2. Experimental Animals and Daily Management

*Macrobrachium rosenbergii* used in the experiment was sourced from the aquaculture farm of the Pearl River Fisheries Research Institute of the Chinese Academy of Fishery Sciences. Prior to commencing the experiment, *M. rosenbergii* were acclimated for 2 weeks to adapt to the experimental environment and were starved for 24 h to empty the gut before sampling. A total of 800 healthy and uniform-sized individuals (with an initial average body weight (BW) of 0.32 ± 0.02 g) were randomly assigned to 16 automatically aerated and recirculating glass tanks (0.6 m × 0.4 m × 0.6 m), with 50 shrimp per tank [34]. There were four groups, with each group containing four replicates. During the rearing period, shrimp were fed twice daily at 9:00 and 18:00. From Weeks 1–10, the daily feeding rate was 7%–9% of BW [36, 37]. From Weeks 11–20, the daily feeding rate was reduced to 5% of BW [38]. Throughout the experimental period, water quality was measured once a week using reagents from Wuxi Aokedan Biotechnology Co., Ltd. (Wuxi, Jiangsu Province, China), the water temperature was maintained at 28–30°C, with a dissolved oxygen content of 6–8 mg L^−1^, a pH range of 7.5–8.2, ammonia nitrogen levels ≤0.2 mg L^−1^, and nitrite levels ≤0.1 mg L^−1^. One to two hours after feeding each morning, feces and uneaten feed were siphoned from the bottom of the tanks. The daily water exchange rate was one-fourth of the tank's total water volume. The aquaculture experiment was conducted in an ornamental fish breeding workshop at the Pearl River Fisheries Research Institute of the Chinese Academy of Fishery Sciences and lasted for 20 weeks, during which the molting cycles of *M. rosenbergii* were recorded.

### 2.3. Sample Collection and Analysis

#### 2.3.1. Sample Collection

Samples were collected in accordance with the animal collection guidelines established by the Animal Experiment Ethics Committee of the Pearl River Fisheries Research Institute (Approval ID: LAEC-PRFRI-2024-03-68), with full respect for animal welfare. At the end of the 10th and 20th weeks of cultivation, after starving the shrimp for 24 h, all *M. rosenbergii* in each tank were weighed, their body length (BL) was measured, and the number of survivors was recorded. From each group, 12 live shrimp were randomly selected for body color measurements. Additionally, 12 shrimp from each group (with tissues from every three-shrimp mixed into one sample, totaling four samples, the same below) were used to collect hemolymph using a 1 mL disposable syringe containing anticoagulant by inserting it along the base of the fifth leg. The hemolymph was then centrifuged at 1000 × *g* for 5 min and the supernatant was collected and stored at −80°C for enzyme activity assays. From each group, the hepatopancreas of 12 shrimp were collected and weighed to calculate the hepatosomatic index (HSI) and assess the expression levels of immune- and antioxidant-related genes. Additionally, at the end of the 20th week of cultivation, eight shrimp from each group were boiled in water for 3 min, removed, and allowed to cool at 25–28°C to measure the body color of the cooked shrimp. Muscle samples were also collected to detect Ax content.

Based on the structural changes in the distal region of the fifth pleopod [39, 40], the molting process of *M. rosenbergii* in each group was divided into intermolt, premolt, and postmolt stages. Four hepatopancreas samples (three shrimp per sample) were collected from each molting stage within each group to assess the differences in the expression levels of molting-related genes.

All hepatopancreas and muscle tissues were frozen in liquid nitrogen and stored at −80°C for subsequent measurement of various indicators.

#### 2.3.2. Growth Indicators Analysis

At the end of the 10th and 20th weeks, the BW, BL, and hepatopancreas weight of *M. rosenbergii* were measured using the tpsDig2 software (F. James Rohlf, Stony Brook University, Stony Brook, NY, USA) and a Mettler Toledo AL-204 precision balance (Mettler Toledo, Inc., Shanghai, China). Growth parameters, including survival rate (SR), WGR, specific growth rate (SGR), and HSI, were calculated using the following formulas:

  $$\begin{array}{c} \text{SR}\left(\%\right)=N_{t}/N_{0}\times 100, \end{array}$$

  $$\begin{array}{c} \text{WGR}\left(\%\right)=\left(W_{t}-W_{0}\right)/W_{0}\times 100, \end{array}$$

  $$\begin{array}{c} \text{SGR}\left(\%/\text{day}\right)=\left(\ln W_{t}-\ln W_{0}\right)/\text{t}\times 100, \end{array}$$

  $$\begin{array}{c} \text{HSI}\left(\%\right)=W_{h}/W_{b}\times 100, \end{array}$$

where *N*_0_ represents the initial number of shrimp (individuals), *N*_t_ represents the final number of shrimp (individuals), *W*_0_ represents the initial BW (g), *W*_t_ is the final BW (g), *t* is the number of days of cultivation (days), *W*_h_ represents the weight of the hepatopancreas (g), and *W*_b_ represents the BW (g).

#### 2.3.3. Body Color Parameters

At the end of the 10th and 20th weeks of cultivation, the *L*^*⁣*^*∗*^^ value (representing brightness, with higher values indicating lighter color), *a*^*⁣*^*∗*^^ value (+*a*^*⁣*^*∗*^^ indicating a reddish hue and −*a*^*⁣*^*∗*^^ indicating a greenish hue), and *b*^*⁣*^*∗*^^ value (+*b*^*⁣*^*∗*^^ indicating a yellowish hue and −*b*^*⁣*^*∗*^^ indicating a bluish hue) of *M. rosenbergii* were measured using a 3nh high-precision colorimeter (Shenzhen Sanlian Technology Co., Ltd., Shenzhen, China). The measurement sites were the first, third, and fifth abdominal segments [28]. Additionally, the colorimetric values of the cooked shrimp were measured at the end of the 20th week of cultivation using the same method. The color difference value, ∆*E*, was calculated using the following formula:

  $$\begin{array}{c} \Delta E=\sqrt{{\left(L^{*}-L_{c}^{*}\right)}^{2}+{\left(a^{*}-a_{c}^{*}\right)}^{2}+{\left(b^{*}-b_{c}^{*}\right)}^{2}}, \end{array}$$

where *L*^*⁣*^*∗*^^ represents the brightness of the treatment group; *L*_c_*⁣*^*∗*^ represents the brightness of the control group; *a*^*⁣*^*∗*^^ represents the redness of the treatment group; *a*_c_*⁣*^*∗*^ represents the redness of the control group; *b*^*⁣*^*∗*^^ represents the yellowness of the treatment group; *b*_c_*⁣*^*∗*^ represents the yellowness of the control group.

#### 2.3.4. Immune and Antioxidant Enzyme Activities Assay

Using the WST-1 method, ammonium molybdate method, thiobarbituric acid (TBA) reaction method, and micro-enzyme labeling method, the activities of superoxide dismutase (SOD; A001-1-2), catalase (CAT; A007-1-1), malondialdehyde (MDA; A003-1-2), and alkaline phosphatase (AKP; A059-2-2) in the hemolymph of *M. rosenbergii* were measured at the end of the 10th and 20th weeks of cultivation, respectively. These measurements were used to evaluate the effects of Ax on the immunity and antioxidant capacity. All operational steps followed the instructions provided in the reagent kits of the Nanjing Jiancheng Bioengineering Institute (Nanjing, China).

#### 2.3.5. Expression Levels of Immune and Molting-Related Genes

Total RNA was extracted from the hepatopancreas using the Trizol method (Invitrogen, Waltham, MA, USA). RNA integrity was assessed using 1% agarose gel electrophoresis and RNA concentration was measured using a Nanodrop-2000 spectrophotometer (Thermo Fisher Scientific Inc., Waltham, MA, USA). cDNA was synthesized using the M-MLV Reverse Transcription Kit (Invitrogen, Waltham, MA, USA) according to the manufacturer's instructions. The primers used for the relevant and reference genes in this study are listed in Table 2. Real-time fluorescent quantitative PCR was performed using a StepOnePlus Real-Time PCR System (Applied Biosystems, Foster City, CA, USA) to assess the expression levels of immune- and molting-related genes in *M. rosenbergii* after the addition of Ax. The reaction system consisted of 1 μL (50 ng μL^−1^) of cDNA, 10 μL of 2 × SYBR Green Premix (Universal), 1 μL of primer (10 pmol μL^−1^), and 7 μL of double-distilled water, totaling 20 μL. The reaction program was as follows: initial denaturation at 95°C for 3 min; 35 cycles of denaturation at 95°C for 40 s, annealing at 60°C for 45 s, and extension at 72°C for 30 s; data collection at 72°C for 10 min, followed by a melting curve obtained by heating at 95°C for 5 s, 60°C for 30 s, and then, 95°C for 15 s.

#### 2.3.6. Measurement of Ax Content

Five grams of muscle were extracted using acetonitrile, defatted with n-hexane, and concentrated. The Ax content in the muscle was determined using high-performance liquid chromatography (HPLC; Shanghai Zhuangrun International Trade Co., Ltd., Shanghai, China), following the method outlined in (SN/T2327-2009).

Briefly, the muscle tissue samples (5.00 ± 0.01 g) were homogenized in 50 mL polypropylene centrifuge tubes containing 30 mL of acetonitrile extraction solvent and 10 g anhydrous sodium sulfate. The mixture was vortex-mixed for 1 min followed by ultrasonic-assisted extraction (water bath for 10 min, 35°C). Subsequent centrifugation (3000 × *g*, 5 min) yielded a supernatant that was transferred to a 125 mL separatory funnel containing 20 mL hexane. After vigorous shaking (30 s) and phase separation, the lower acetonitrile layer was collected. This extraction protocol was repeated twice for the residual pellet.

The combined acetonitrile fractions obtained above were supplemented with 5 mL 1-propanol and concentrated to near-dryness via rotary evaporation (<40°C). The residue was reconstituted in acetonitrile with volumetric adjustment to 5.0 mL, followed by membrane filtration (0.20 μm of pore size). Quantitative analysis of Ax was performed using HPLC (Shanghai Zhuangrun International Trade Co., Ltd., Shanghai, China).

### 2.4. Statistical Analysis

Using GraphPad Prism 9 software (GraphPad Software, San Diego, CA, USA), a difference analysis was conducted on growth indicators, colorimetric values, Ax content, immune and antioxidant parameters, and related gene expression levels across various groups. Data are presented as “mean ± standard error (SE).” Significant differences were indicated by *p* < 0.05 and extremely significant differences were indicated by *p* < 0.01.

## 3. Results

### 3.1. Growth Performance

The effects of various Ax supplementation levels on the growth indicators of the *M. rosenbergii* are shown in Table 3. The growth indicator results at Week 10 showed that the BW of the Ax200 group was significantly higher than that of the Ax100 group and the control group (*p* < 0.05). Furthermore, the Ax50 group demonstrated a significant increase in BW compared to the control group (*p* < 0.05); however, no significant difference was observed between the Ax50 and Ax100 groups. Both the Ax50 and Ax100 groups exhibited significantly higher rates of weight gain than the control group (*p* < 0.05), although no significant difference was observed when compared to the Ax200 group. The SGRs of all three Ax groups (Ax50, Ax100, and Ax200) were significantly higher than that of the control group (*p* < 0.05). The HSI of the Ax200 group was significantly lower than that of the control group (*p* < 0.05). There were no significant differences in the SRs among the groups.

The growth of *M. rosenbergii* at Week 20 indicated that the BWs of the three groups fed with Ax were significantly greater than those of the control group that did not receive Ax (*p* < 0.05). Regarding BL, the control group was significantly shorter than in the Ax100 group (*p* < 0.05) and the Ax200 group (*p* < 0.01). Both the Ax50 and Ax100 groups showed significantly higher rates of weight gain than the control group (*p* < 0.05), with no significant differences observed between these groups and the Ax200 group. The SGRs of the Ax100 and Ax200 groups were significantly higher than those of the control group and Ax50 group (*p* < 0.05). In terms of SR, the Ax50 group exhibited a significantly higher SR compared to the other three groups (*p* < 0.05). There were no significant differences in the hepatosomatic indices between the groups. Additionally, the molting cycle in the Ax100 group was significantly shorter than that in the control group (*p* < 0.05).

### 3.2. Body Color and Ax Content in Muscle

**Figure 1:**
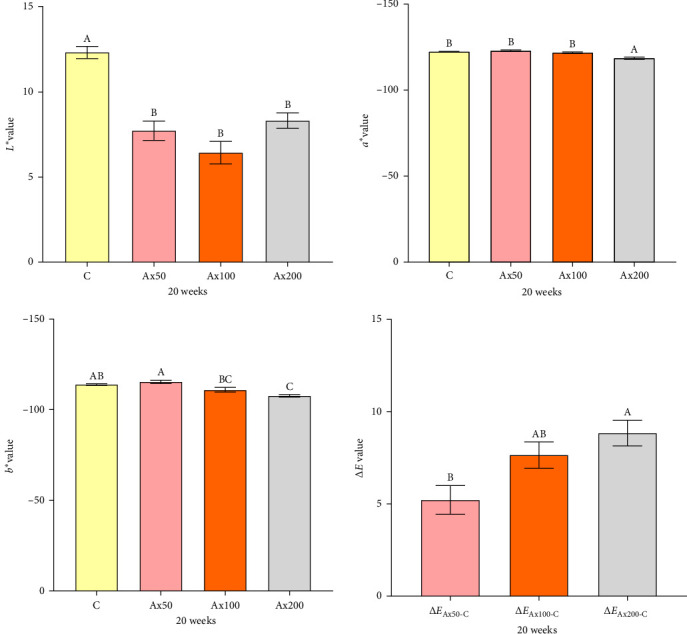
Color values (*L*^⁣^*∗*^^, *a*^⁣^*∗*^^, *b*^⁣^*∗*^^, and Δ*E*) of live *M. rosenbergii* after 20 weeks of cultivation. *L*^⁣^*∗*^^: lightness; *a*^⁣^*∗*^^: redness; *b*^⁣^*∗*^^: yellowness. Data are presented as mean ± SEM (*n* = 12). The bars with different letters are significantly different (*p＜*0.01).

**Figure 2:**
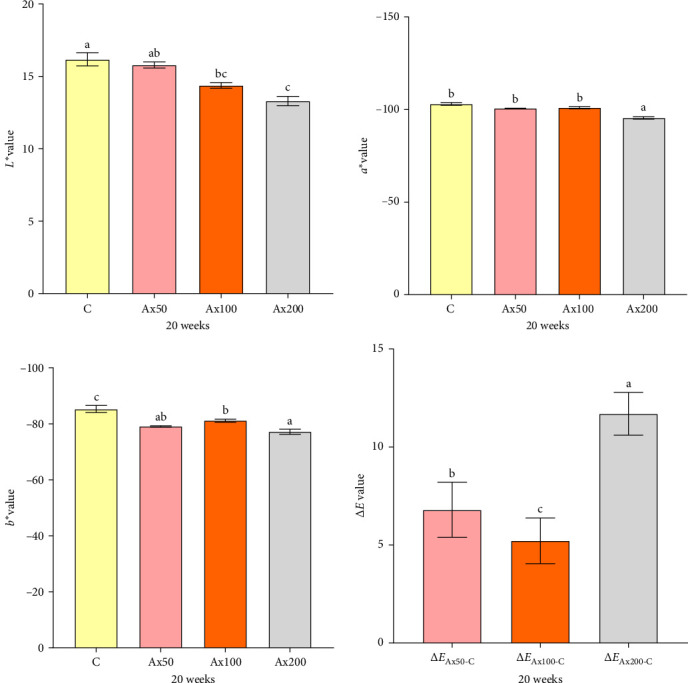
Color values (*L*^⁣^*∗*^^, *a*^⁣^*∗*^^, *b*^⁣^*∗*^^, and Δ*E*) of cooked *M. rosenbergii* after 20 weeks of cultivation. *L*^⁣^*∗*^^: lightness; *a*^⁣^*∗*^^: redness; *b*^⁣^*∗*^^: yellowness. Data are presented as mean ± SEM (*n* = 12). The bars with different letters are significantly different (*p＜*0.05).

**Figure 3:**
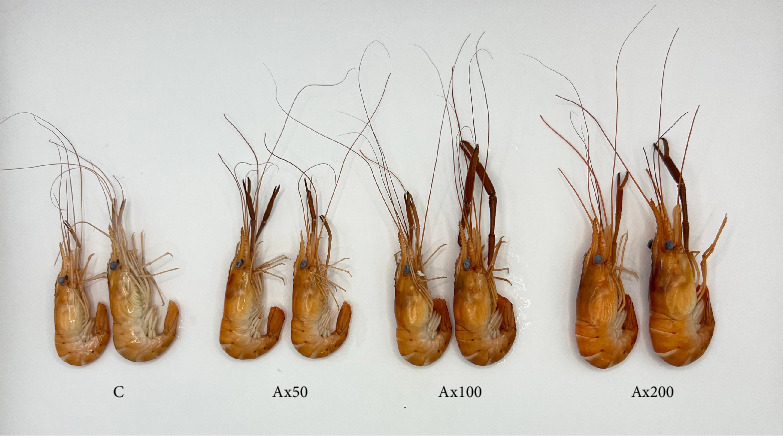
The appearance of cooked *M. rosenbergii* after 20 weeks of being fed with experimental diet (*n* = 12 for each group).

Body color results of live *M. rosenbergii* at Week 10 with different levels of Ax supplementation are presented in Figure S1 of the Supporting Information. The *L*^*⁣*^*∗*^^ value of the group without Ax supplementation was extremely significantly higher than that of the Ax supplemented groups (*p* < 0.01). Conversely, the *a*^*⁣*^*∗*^^ and *b*^*⁣*^*∗*^^ values of the Ax100 and Ax200 groups were extremely significantly higher than those of the remaining groups (*p* < 0.01). However, the Δ*E* values indicated no significant differences in color variation among the *M. rosenbergii* fed with Ax. The body color of live shrimp at Week 20 is shown in Figure 1. The *L*^⁣^*∗*^^ values of all the treatment groups were extremely significantly lower than those of the control group (*p* < 0.01). The *a*^*⁣*^*∗*^^ value of the Ax200 group was strongly significantly higher than those of the other three groups (*p* < 0.01). Additionally, the *b*^*⁣*^*∗*^^ values for the Ax100 and Ax200 groups were extremely significantly greater than those of the control group (*p* < 0.01), and the Ax200 group exhibited an extremely significant increase compared with the Ax50 group (*p* < 0.01). The Δ*E* values for the higher-dose group were significantly higher than that of for the lower-dose group (Δ*E*_Ax50-C_; *p* < 0.01), with no significant difference between the two lower-dose groups (Δ*E*_Ax50−*C*_ and Δ*E*_Ax100−*C*_). The body color of cooked *M. rosenbergii* at Week 20 with different levels of Ax supplementation is shown in Figure 2. As the dosage of Ax in the base feed increased, the color of the cooked *M. rosenbergii* gradually intensified in Week 20 (Figure 3).

**Figure 4:**
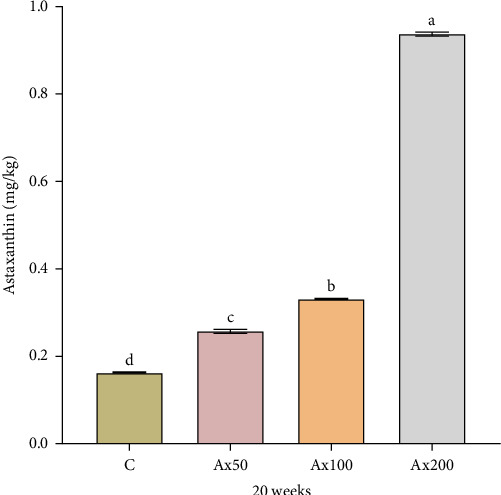
Contents of astaxanthin in muscle of *M. rosenbergii* after 20 weeks of being fed with experimental diet. Data are presented as mean ± SEM (*n* = 12). The bars with different letters are significantly different (*p＜*0.05).

Figure 4 shows the Ax content in the muscle of *M. rosenbergii* at the 20th week, with varying levels of Ax supplementation. Following the addition of Ax, the muscle content increased significantly in a stepwise manner compared to that of the control group (*p* < 0.05).

### 3.3. Immune and Antioxidant Capabilities in the Hemolymph

**Figure 5:**
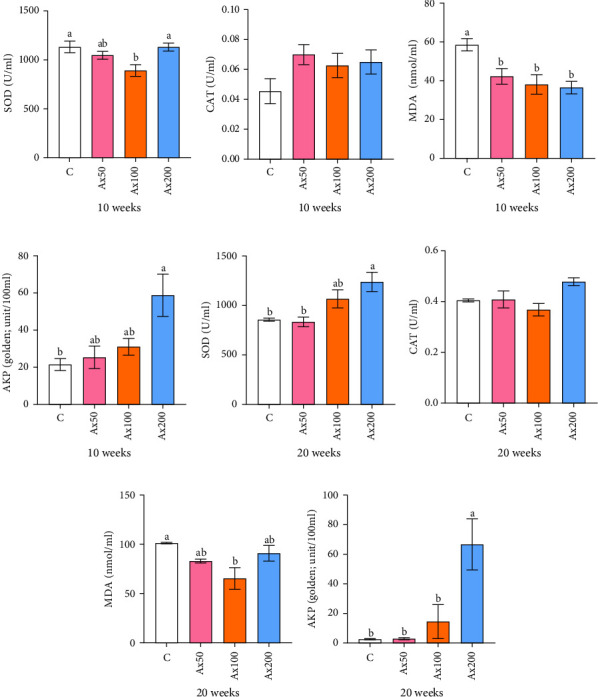
Effects of dietary supplemented with astaxanthin on immunity and antioxidant enzyme activities of the hemolymph of *M. rosenbergii* after 10 (A–D) and 20 (E–H) weeks of cultivation. AKP, alkaline phosphatase; CAT: catalase; MDA: malondialdehyde; SOD: superoxide dismutase. Data are presented as mean ± SEM (*n* = 12). The bars with different letters are significantly different (*p＜*0.05).

The effects of Ax supplementation on the immune and antioxidant enzyme activities in the hemolymph of *M. rosenbergii* were evaluated (Figure 5). At Week 10 of cultivation, the enzyme activity results showed that the SOD activity in the Ax100 group was significantly lower than that in both the control and Ax200 groups (*p* < 0.05; Figure 5A). Additionally, MDA levels in the Ax-fed groups were significantly lower than those in the control group (*p* < 0.05; Figure 5C). AKP activity in the Ax200 group was significantly higher than that in the control group (*p* < 0.05; Figure 5D). However, no significant differences in CAT activity were observed between groups (Figure 5B). At Week 20 of cultivation, the enzyme activity results revealed that SOD activity in the Ax200 group was significantly higher than that in both the control and Ax50 groups (*p* < 0.05), whereas SOD activity in the Ax100 group did not differ significantly from that in the other three groups (Figure 5E). Furthermore, MDA levels in the Ax100 group were significantly lower than those in the control group (*p* < 0.05; Figure 5G). AKP activity in the Ax200 group was significantly higher than in the other three groups (*p* < 0.05; Figure 5H). However, there were no significant differences in CAT activity between the groups (Figure 5F).

### 3.4. Immune and Antioxidant Gene Expression

**Figure 6:**
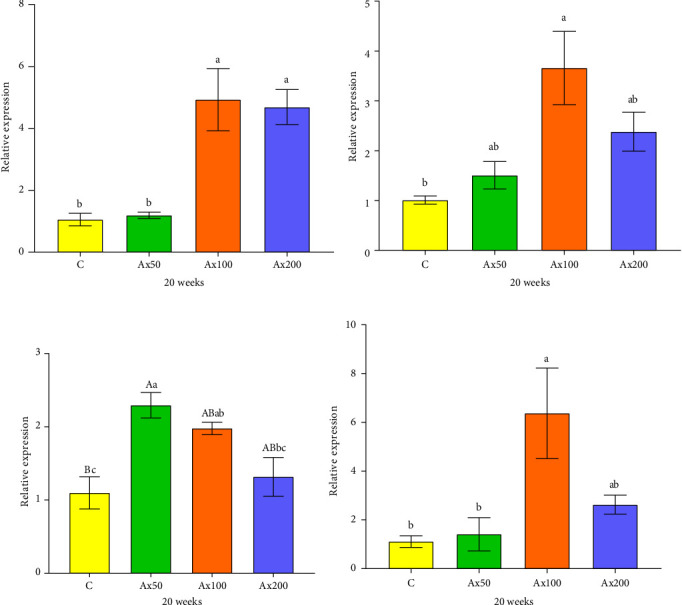
Effects of diet supplemented with astaxanthin on the expressions of immune and antioxidant genes in the hepatopancreas of *M. rosenbergii* after 20 weeks (A–D). Data are presented as mean ± SEM (*n* = 12). The bars with the different uppercase letter indicate extremely significant differences (*p＜*0.01), bars with different lowercase letters are significantly different (*p* < 0.05). (A) *sod*, (B) *cat*, (C) *gsh-px*, and (D) *acp*.

The expression levels of immune and antioxidant genes in the hepatopancreas at Week 20 are shown in Figure 6. The expression levels of the *sod* gene in the Ax100 and Ax200 groups were significantly higher than those in the control and Ax50 groups (*p* < 0.05). The expression level in *cat* gene in the Ax100 group was significantly higher than that in the control group (*p* < 0.05). The expression level of the *gsh-px* gene in the control group was significantly lower than that in the Ax100 group (*p* < 0.05) and extremely significantly lower than that in the Ax50 group (*p* < 0.01), while the Ax50 group had a significantly higher expression level than the Ax200 group (*p* < 0.05). The expression level of the *acp* gene in the Ax100 group was significantly higher than that in the control and Ax50 groups (*p* < 0.05).

### 3.5. Molting-Related Gene Expression

**Figure 7:**
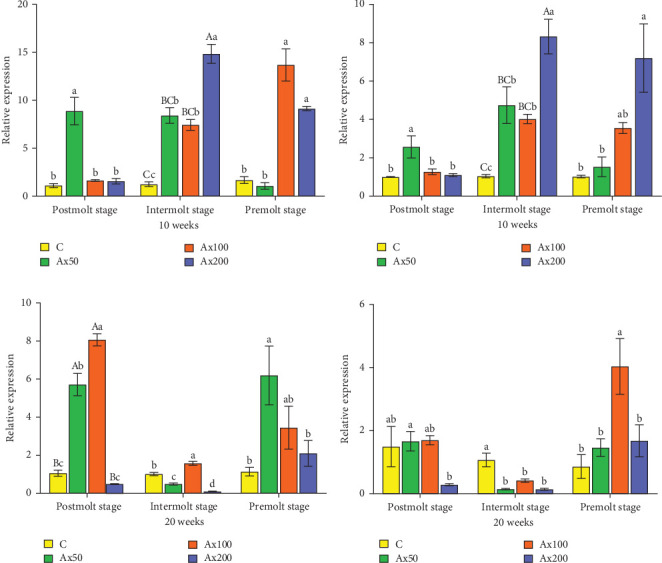
Effects of diet supplemented with astaxanthin on the expressions of molting genes in the hepatopancreas at different molting periods of *M. rosenbergii* at 10 and 20 weeks. *EcR*, ecdysone receptor; *RXR*, retinoid X receptor. Data are presented as mean ± SEM (*n* = 12). The bars with the different uppercase letter indicate extremely significant differences (*p* < 0.01), bars with different lowercase letters are significantly different (*p* < 0.05). (A, C) *ECR* and (B, D) *RXR*.

The results of ecdysone gene expression levels at different molting stages of *M. rosenbergii* fed Ax for 10 weeks are shown in Figure 7. During the postmolt stage, the ecdysteroid receptor (*EcR*) gene expression level in the Ax50 group was significantly higher than that in the other three groups (*p* < 0.05). In the intermolt stage, the Ax200 group showed significantly higher expression than the two lower dose groups (*p* < 0.05) and the unfed group (*p* < 0.01). During the premolt stage, the Ax100 and Ax200 groups showed significantly higher expression levels than the low-dose and unfed groups (*p* < 0.05). Regarding retinoid X receptor (*RXR*) gene expression, the postmolt stage exhibited significantly higher gene expression levels in the Ax50 group than in the other three groups (*p* < 0.05). In the intermolt stage, the high-dose group showed significantly higher expression than the two lower dose groups (*p* < 0.05) and extremely significantly higher expression than the control group (*p* < 0.01). During the premolt stage, the high-dose group showed significantly higher expression levels than the control group and Ax50 group (*p* < 0.05).

Gene expression results after 20 weeks: During the postmolt stage, the *EcR* gene expression level in the Ax100 group was significantly higher than that in the Ax50 group (*p* < 0.05) and was significantly higher than that in the other two groups (*p* < 0.01). In the intermolt stage, the Ax100 group showed significantly higher expression than the other three groups (*p* < 0.05). During the premolt stage, gene expression levels in the Ax50 group were significantly higher than those in the control and Ax200 groups (*p* < 0.05), with no significant differences among the other three groups. For the *RXR* gene: During the postmolt stage, the gene expression level in the Ax50 group was significantly higher than that in the Ax200 group (*p* < 0.05). At the intermolt stage, the gene expression levels in the control group were significantly higher than those in the other three groups (*p* < 0.05). During the premolt stage, the gene expression level in the Ax100 group was significantly higher than that in the other groups (*p* < 0.05).

## 4. Discussion

### 4.1. Growth Performance and Molting

Numerous studies have reported significant improvements in the growth performance of crustaceans owing to Ax. For example, feeding *P. clarkii* diets supplemented with 0.6% (containing 126.60 mg kg^−1^ of Ax) and 1.2% (containing 253.20 mg kg^−1^ of Ax) *Haematococcus pluvialis* resulted in a significantly higher final BW, WGR, and SGR compared to the basal diet group [20]. Additionally, feeding *L. vannamei* diets containing 160 mg kg^−1^ of Ax increased their weight gain and SGRs by 71.86% and 0.17%, respectively [21]. Our study indicated that the addition of Ax improved both the WGR and SGR of larvae, especially with a feeding level of 100 mg kg⁻^1^ of Ax. However, inconsistent with our study, Xu et al. [35] found that feeding *M. rosenbergii* diets supplemented with 100 and 200 mg kg^−1^ of Ax for 10 weeks led to a slight, but not significant, increase in the WGR and SGR compared with the control group. We speculate that the differences may be due to variations in the composition of the basal diets and differences in feeding patterns [25, 35]. Studies on *P. monodon* [41] and *Marsupenaeus japonicus* [42] have demonstrated that the addition of Ax can improve weight gain and SGRs. The result of HSI showed that a 200 mg kg^−1^ Ax supplementation significantly reduced HSI by Week 10. The addition of higher level of Ax may have contributed to enhanced consumption or translocation of nutrients within the hepatopancreas of *M. rosenbergii* [43]. However, excessive Ax (320 mg kg^−1^) decreased partial functions of antioxidant index, suggesting that there may be a dose-dependent threshold for the absorption of Ax in feed by prawns [35]. Furthermore, feeding moderate crustaceans Ax can also benefit changes in their molting frequency. For instance, the addition of 80 mg kg^−1^ Ax has been shown to increase the molting frequency and shorten the molting cycle of *L. vannamei* [29]. This study also demonstrated that the addition of Ax can shorten the molting interval of *M. rosenbergii*, particularly with the addition of 100 mg kg^−1^ of Ax. Based on subsequent gene detection results for *EcR* and *RXR*, it is hypothesized that the incorporation of Ax may have enhanced the upregulation of ecdysterone and ecdysis-related genes by the EcR–RXR complex, resulting in a decreasing in molting interval and accelerated growth in *M. rosenbergii* [44].

### 4.2. Body Color and Ax Content

The body color of aquatic animals can vary depending on pigment content and dietary intake. Ax is commonly used in aquaculture to promote pigmentation, alter the body color of aquatic organisms and subsequently boost the product quality and price [45, 46]. Body color indices typically include brightness, redness, and yellowness [47, 48]. In vertebrates, when *Plectropomus leopardus* [49] and *Blood parrotfish* [50] were fed diets supplemented with 0.2% *Haematococcus lacustris* algae (containing 3% Ax) and 0.45 g kg^−1^ of Ax, respectively, there was a significant decrease in brightness for both species, while both redness and yellowness increased significantly. In crustaceans, the addition of Ax to feed can improve the brightness, redness, and yellowness of species such as *Penaeus japonicus* [42] and *P. monodon* [51]. Similarly, this study indicated that the addition of Ax decreased the brightness and increased the redness and yellowness values of *M. rosenbergii* (both live and cooked). The color intensity of cooked *M. rosenbergii* increased as the amount of Ax added to the base feed increased. Moreover, the level of astaxanthin present in muscle tissue is regarded as a key indicator for assessing the utilization efficiency of Ax within organisms [52]. Our results showed that Ax content in the muscle of *M. rosenbergii* significantly increased with the addition of Ax. This finding is similar to that observed in *Pagrus pagrus* [53], *P. monodon* [54], and *L. vannamei* [21], suggesting homogeneity in the accumulation of Ax in aquatic animals.

### 4.3. Immunity and Antioxidant Enzyme Activity

Crustaceans lack a vertebrate immune system and can only resist pathogen invasion through a nonspecific immune system [55, 56]. AKP level is an important parameter for assessing the nonspecific immunity in crustaceans. As a nonspecific phosphohydrolase, it regulates the metabolic activities of aquatic animals by catalyzing the hydrolysis of phosphate monoesters and transfer of phosphate groups [57]. Additionally, AKP enhances stress resistance by increasing its activity [58] and addition of Ax to feed can alter AKP activity in crustaceans. For instance, supplementing Ax in the feed of *P. clarkii* [58] and juvenile *Cherax quadricarinatus* [59] significantly increases the AKP activity in their hemolymph, indicating an enhancement in their immune performance. Here, the addition of Ax to the feed also increased the AKP activity of *M. rosenbergii*, indicating that supplementing feed with Ax can enhance the immune capability of *M. rosenbergii*. In a similar study, the addition of Ax (100 and 200 mg kg^−1^) to the feed of *M. rosenbergii* resulted in a decrease in AKP activity after 10 weeks of feeding, which is inconsistent with the results of this study [35]. Combined with findings from studies on *P. clarkii* [20, 58] and *L. vannamei* [21], it is hypothesized that Ax can enhance AKP activity and the immune capability of *M. rosenbergii*.

Additionally, the addition of Ax to feed can alter the antioxidant capacity of organisms. SOD, CAT, and MDA are important indicators of the antioxidant capacity of organisms [60–62]. This study found that the addition of high-dose Ax (200 mg kg^−1^) could enhance SOD activity, while there was no significant difference in CAT activity. Similarly, it was found that adding Ax to the feed for 15 days significantly increases SOD activity in the hepatopancreas, whereas there was no significant difference in CAT activity in *L. vannamei* [63]. Surprisingly, the SOD activity was lower in Ax100 group than that of control group at Week 10, speculating that other antioxidant pathways may be involved, but the exact mechanism needs to be further investigated. SOD orchestrates the critical dismutation of superoxide anion radicals (O_2_^−^) into hydrogen peroxide (H_2_O_2_) and molecular oxygen (O_2_), constituting the primary antioxidant defense mechanism [64, 65]. Subsequently, H_2_O_2_ undergoes enzymatic detoxification via CAT or glutathione peroxidase (GPx), yielding water (H_2_O), and thereby effectively mitigating reactive oxygen species (ROS) accumulation while preserving cellular integrity against oxidative damage [66–68]. Considering the concurrent upregulation of the *gsh-px* gene, we posit that the observed stability in CAT activity may arise from a compensatory surge in GPx functionality. This enhanced GPx capacity appears sufficient to maintain intracellular redox homeostasis, thereby obviating the requirement for substantial compensatory modulation of CAT activity. MDA is a reactive product of lipid peroxidation, an increase of which indicates heightened damage to the body, whereas a decrease suggests enhanced antioxidant capacity [58, 69, 70]. The addition of *H. pluvialis* (containing Ax) to the feed of *L.vannamei* resulted in a decrease in MDA activity in the hemolymph, indicating that Ax can enhance the antioxidant capacity of *L. vannamei* [37]. After 20 weeks of feeding with Ax, a reduction in MDA activity was observed in the hemolymph of *M. rosenbergii*, with a significant decrease particularly at the addition level of 100 mg kg^−1^. This demonstrated that the inclusion of Ax can improve the antioxidant capacity of *M. rosenbergii*, particularly in the presence of high-dose Ax.

### 4.4. Immunity, Antioxidant, and Molting Related Gene Expression

Immune- and antioxidant-related genes play crucial roles in regulating the immune and antioxidant capacities of aquatic animals [59]. In particular, the genes such as *sod*, *cat*, *gsh-px*, and *acp*, which are associated with immunity and antioxidant defenses, are frequently used to assess the strength of an organism's immune and antioxidant capabilities [71, 72]. The increased expression levels of *sod*, *cat*, and *gsh-px*, found in the spleen of rainbow trout, demonstrate an enhancement in the species' immune and antioxidant capabilities [73]. The expression levels of immune- and antioxidant-related genes are influenced by numerous factors, such as temperature [74], ammonia nitrogen [75], and nutrition [72]. It has been reported that the use of Ax in aquatic animals can also alter the expression levels of such genes [76]. This study discovered that diets supplemented with Ax elevated the expression levels of immune and antioxidant-related genes (*sod*, *cat*, *gsh-px*, and *acp*) in *M. rosenbergii*. Diets containing Ax have also been shown to elevate the expression levels of the *sod* and *acp* genes in *Babylonia areolata* [77], while decreasing the expression levels of the *sod*, *cat*, and *gsh-px* genes in *L. vannamei* [37]. It is notable that excessive addition of Ax has a negative effect on antioxidant capacity [35], which may be the explanation for the significantly lower of gsh-px expression level in Ax200group compared to the Ax50 group in the present study.

The molting of crustaceans is classified into growth molting and reproductive molting and is regulated by molting-related hormones and their associated genes, such as ecdysteroids and *EcR* and *RXR* genes [78–81]. Both EcR and RXR are members of the nuclear receptor superfamily, where EcR specifically binds to ecdysteroids and RXR functions as a ligand activator. Furthermore, the EcR/RXR heterodimer complex, formed by the dimerization of *EcR* and *RXR* genes, is involved in the initiation of molting [82, 83]. In this work, the expression trends of the *EcR* and *RXR* genes differed across various supplementation levels of Ax and molting stages both at Weeks 10 and 20. These results indicate that changes in the expression levels of molting-related genes in crustaceans are complex and vary with different Ax supplementation levels and molting stages. Further research is required to investigate the molecular mechanisms underlying molting regulated by dietary Ax in crustaceans using a transcriptome-wide analysis or pathway-level study.

## 5. Conclusion

This study indicated that the addition of Ax can enhance the growth performance of *M. rosenbergii*, particularly when supplemented at 100 mg kg^−1^, where significant increases in weight gain and SGR were observed, along with a notable shortening of the molting cycle. Concurrently, Ax enhanced the redness and yellowness of *M. rosenbergii* (both live and cooked), and the Ax content in the muscle increased with the addition of Ax to the feed. Furthermore, astaxanthin improved the immune and antioxidant capabilities and affected the expression levels of related molting genes, implying the Ax content can regarded as one of the biochemical indicators for assessing the organism's health status. Therefore, Ax had better effect on the growth, molting, immunity, and antioxidant capacity of *M. rosenbergii* when supplemented with 100 mg kg^−1^ of Ax in the basal diet. The finding will provide reference for the application of Ax as a functional feed supplement in commercial prawn farming.

## Figures and Tables

**Table 1:** Ingredients and proximate compositions of the diet (dried mass basis).

Ingredients (g kg^−1^)	Astaxanthin (Ax) supplementation level (mg kg^−1^ diet)			
	C	Ax50	Ax100	Ax200
Basic diets^a^	1000	998.33	996.67	993.33
Ax source^b^	0	1.67	3.33	6.67
Total	1000	1000	1000	1000
Proximate compositions				
Crude protein (%)	49.91	49.36	49.52	48.94
Crude lipid (%)	4.81	6.17	5.22	5.83
Ash (%)	13.66	13.66	13.5	13.33
Moisture (%)	5.19	4.34	4.55	4.76

*Note:* C, control diet without Ax supplementation; Ax50, diet with 50 mg kg^−1^ natural Ax; Ax100, diet with 100 mg kg^−1^ natural Ax; Ax200, diet with 200 mg kg^−1^ natural Ax.

^a^Basic diets: Vitality Treasure Feed Co., Ltd., Zhuhai, Guangdong, China.

^b^Ax source: *Haematococcus pluvialis* powder containing 3% Ax made by Aifumei Biotechnology Co., Guangzhou, China, was used as natural Ax.

**Table 2:** qRT-PCR primers used in the study.

Primer name	Sequence (5′–3′)		Sources
*cat*	F	ACTTCATTACCCTGAGACCCG	HQ668089.1
	R	TTTCCCTCAGCATTGACCAG	
*acp*	F	GTTTACACTCGCCTTATCCTCCG	KT765023.1
	R	CTTTGTGCATGAACATGACCCTG	
*sod*	F	GTGGCTGGGACAATCGTTT	DQ121374.1
	R	GTCTTATTTCGGCATCAGGC	
*gsh-px*	F	AGGGAAGGTGATTCTTGTGGA	FJ670566.1
	R	TTACAGGGGAAAGCCAGGA	
*RXR*	F	GATCGGCAGTCCCCTTTGAA	MZ501612.1
	R	TTGGACACACTGGGAGAAGC	
*EcR*	F	ACAGTTCAGCTCATAGTGGA	This study
	R	CTCTCAGCATCATCACTTCG	
*β*-actin	F	CAGGGAAAAGATGACCCAGA	AY626840
	R	GGAAGTGCATACCCCTCGTA	

**Table 3:** Effects of dietary astaxanthin levels on growth performance of *M. rosenbergii*.

Item	C	Ax50	Ax100	Ax200
BW (g)				
0 W	0.34 ± 0.07	0.30 ± 0.06	0.29 ± 0.07	0.34 ± 0.05
10 W	4.37 ± 0.95^c^	5.19 ± 0.74^ab^	4.88 ± 0.60^bc^	5.55 ± 0.88^a^
20 W	8.46 ± 0.56^b^	10.12 ± 0.56^a^	10.80 ± 0.51^a^	10.75 ± 0.72^a^
BL (cm)				
0 W	3.33 ± 0.07	3.18 ± 0.13	3.45 ± 0.08	3.31 ± 0.07
10 W	7.55 ± 0.20^b^	8.06 ± 0.38^ab^	7.81 ± 0.27^ab^	8.29 ± 0.20^a^
20 W	8.02 ± 0.29^Bc^	8.73 ± 0.15^ABbc^	9.47 ± 0.33^ABab^	9.96 ± 0.23^Aa^
WGR (%)				
10 W	1175 ± 16.96^b^	1652 ± 176.00^a^	1610 ± 258.20^a^	1527 ± 39.17^ab^
20 W	2399.00 ± 119.50^b^	3334.74 ± 206.70^a^	3716.44 ± 383.00^a^	3068.00 ± 147.90^ab^
SGR (%/day)				
10 W	3.64 ± 0.02^b^	4.09 ± 0.14^a^	4.05 ± 0.20^a^	3.99 ± 0.03^a^
20 W	2.29 ± 0.03^b^	2.52 ± 0.05^b^	5.18 ± 0.13^a^	4.93 ± 0.07^a^
SR (%)				
10 W	51.50 ± 5.19	69.50 ± 7.89	50.50 ± 13.79	49.50 ± 5.74
20 W	36.91 ± 0.43^b^	47.50 ± 0.66^a^	37.88 ± 0.78^b^	37.95 ± 0.46^b^
HSI (%)				
10 W	6.13 ± 0.78^a^	6.02 ± 0.51^ab^	5.87 ± 0.32^ab^	5.26 ± 0.36^b^
20 W	5.34 ± 0.34	4.94 ± 0.24	5.17 ± 0.33	6.00 ± 0.46
Molt cycle (days)	7.55 ± 0.12^a^	7.04 ± 0.16^ab^	6.79 ± 0.13^b^	6.85 ± 0.17^ab^

*Note:* Data are presented as mean ± SEM (*n* = 20). Mean values within a row with different uppercase superscript letters indicate highly significant differences (*p* < 0.01), and those with different lowercase superscript letters indicate significant differences (*p* < 0.05).

Abbreviations: 10 W, Week 10; 20 W, Week 20.

## Data Availability

The data that support the findings of this study are available from the corresponding author upon reasonable request.

## Supporting Information

Supporting Information Figure S1. The color values (*L⁣*^*∗*^, *a⁣*^*∗*^, *b⁣*^*∗*^, and *ΔE*) of live M. rosenbergii after 10 weeks of cultivation. *L⁣*^*∗*^: lightness; *a⁣*^*∗*^: redness; *b⁣*^*∗*^: yellowness. Data are presented as mean ± SEM (*n* = 12). Bars with the different uppercase letter indicate extremely significant differences (*p* < 0.01).
