# Supplementary figures and images for: Effects of Dietary Astaxanthin on Growth, Coloration, Immunity, and Antioxidant Capacity in Macrobrachium Rosenbergii
Source: Aquac Nutr. 2025 Jun 13;2025:8865839. doi: 10.1155/anu/8865839 (PMC12181043; doi:10.1155/anu/8865839)

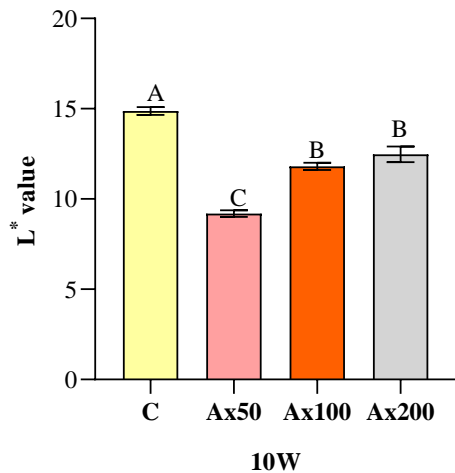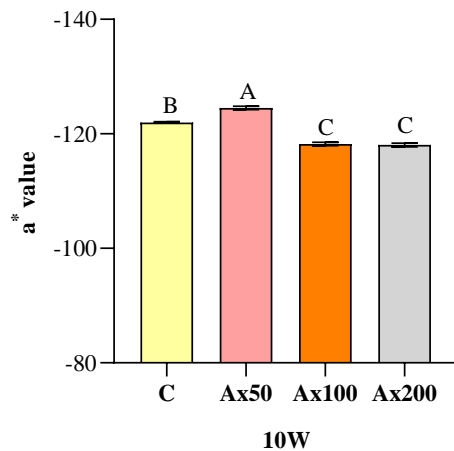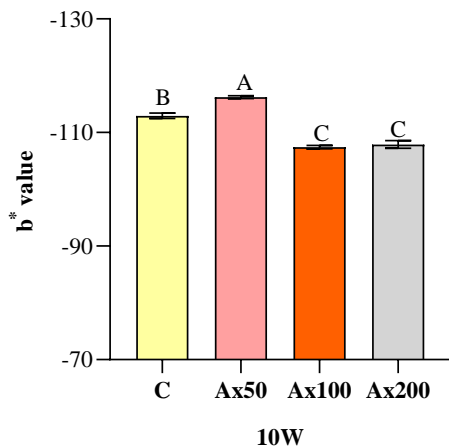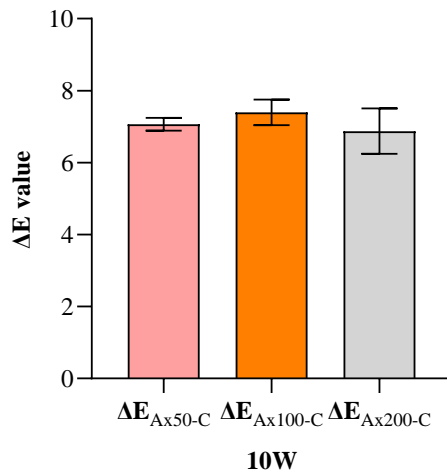

Supplement: Supporting Information — Figure S1. The color values (L⁣∗, a⁣∗, b⁣∗, and ΔE) of live M. rosenbergii after 10 weeks of cultivation. L⁣∗: lightness; a⁣∗: redness; b⁣∗: yellowness. Data are presented as mean ± SEM (n = 12). Bars with the different uppercase letter indicate extremely significant differences (p < 0.01). [file 8865839.f1.pdf]
